# Social capital and health information seeking in China

**DOI:** 10.1186/s12889-022-13895-2

**Published:** 2022-08-10

**Authors:** Qianfeng Lu, Angela Chang, Guoming Yu, Ya Yang, Peter J. Schulz

**Affiliations:** 1 Faculty of Communication, Culture and Society, Università Della Svizzera Italiana, Via Buffi 13, 6900 Lugano, Switzerland; 2 Faculty of Social Sciences, University of Macau, Macau, China; 3 School of Journalism and Communication, Beijing Normal University, Beijing, China; 4 Department of Communication & Media, Ewha Womans University, Seoul, South Korea

**Keywords:** Social capital, Social support, Social networks, Health information-seeking behavior

## Abstract

**Background:**

People’s potentials to seek health information can be affected by their social context, such as their social networks and the resources provided through those social networks. In the past decades, the concept of social capital has been widely used in the health realm to indicate people’s social context. However, not many such studies were conducted in China. Chinese society has its special quality that many Western societies lack: people traditionally render strong value to family relations and rely heavily on strong social ties in their social life. Therefore, the purpose of this study was to examine the association between different types of social capital and health information-seeking behavior (HISB) in the Chinese context. The different types of social capital were primarily bonding and bridging, as well as cognitive and structural ones.

**Methods:**

Our analysis is based on a total of 3090 cases taken from the Health Information National Trends Survey (HINTS) – China, 2017. Dataset was weighted due to the overrepresentation of female respondents and hierarchical multiple regression analyses as well as binary logistic regression tests were operated to examine the associations between people’s social capital and their HISB.

**Results:**

Some aspects of social capital emerged as positive predictors of HISB: information support (standing in for the cognitive component of social capital) promoted health information seeking, organization memberships (standing in for the structural component) encouraged cancer information seeking, and both the use of the internet and of traditional media for gaining health information were positively linked with bridging networks and organization memberships. Bonding networks (structural component) were not correlated with any other of the key variables and emotional support (cognitive social capital) was consistently associated with all health information-seeking indicators negatively.

**Conclusions:**

Social capital demonstrated significant and complex relationships with HISB in China. Structural social capital generally encouraged HISB in China, especially the bridging aspects including bridging networks and organization memberships. On the other hand, emotional support as cognitive social capital damaged people’s initiatives in seeking health-related information.

**Supplementary Information:**

The online version contains supplementary material available at 10.1186/s12889-022-13895-2.

## Background

### The potential of health information seeking

Health information is among the most-sought subject matters on the internet. Situations in which people seek such information can be easily imagined, e.g. we cannot decide whether we need to see a doctor or can help ourselves with new symptoms; or we need arguments because we intend to challenge our doctor’s diagnosis or treatment suggestion. Improvements in technology, especially the development of the internet, have dramatically eased health information-seeking behavior (HISB). People are exposed to diverse and easily accessible information channels [1, 2], and they use them [3].

Health information-seeking affects people’s health in many ways. In the context of prevention, information can potentially affect people's attitudes and beliefs towards certain health behaviors and motivate individuals to change their behavior in a health-serving way [4]. It also functions as a coping strategy in dealing with health-threatening situations [5], enhancing people’s understanding of their health, illnesses and related challenges [6]. In particular, HISB has become an essential means for patients to gain health knowledge they need to join their physician in patient-collaborated medical care, the current ideal for doctor-patient communication [7]. Also, HISB creates in people a feeling of control and releases uncertainty-related emotions such as anxieties [8, 9].

Seeking health information has become an option in many situations, and the motives to do it are now an important subject for health communication research. On balance, HISB has favourable health consequences, but many associations are unexplored so far. This article is concerned with one of the antecedents of HISB: social capital; in particular we focus on Chinese populations. In the remainder of this background, we will address the questions: why this concept, social capital, and why this country? The remarks above should answer the question: why study HISB?

Our observations and analyses are based on a few given trends, which provide a background. The availability of health information was just described, and we should be aware that the growth in digital health information has not only expanded and accelerated the information flow, but given it a completely new quality [10, 11]. The second given is the modernization of China, in the progress of which some valuable things were lost, and some treasures found. A sure loss concerns the tight social bonds within families and among neighbors [12, 13]. Modernization lead to a sure loss concerning the tight social bonds with families and among neighbors, while it created new functions to be filled by institutions or individuals. An example can be found in the way of seek health information. The main information sources were dominated by interpersonal channels such as family, friends and health experts, while people nowadays are exposed to much more information acquisition tools and means. People shares their medical experience, raise up health questions and seek for or provide others with social support on the Internet; public institutes broadcast health knowledge and policy online.

### Social capital

Social capital refers to the relationships of an individual or organization to other individuals or organizations; the relationships are resources which, if used properly, can lead to the development and accumulation of capital in the classic sense [14]. The model can easily be imagined with health as the outcome. Social capital has become an exceptionally wide and successful term. It serves as an umbrella term containing many different concepts [15], three of which are to be found in most definitions: social networks, norms of reciprocity, and trust [16, 17]. Putnam (1995) defined social capital as a combination of these three main elements: "features of social organization such as networks, norms, and social trust that facilitate co-ordination and co-operation for mutual benefit (p. 67).” The underlying idea states that people’s social networks and associated reciprocities have value [18].

Social capital has been shown to promote people's physical and mental health [19–21]. It also affects individuals’ health-related behaviors including alcohol consumption, diet, cigarette smoking, physical exercises and HISB [22–26]. It influences people's health through several mechanisms, e.g., by providing individuals’ tangible benefits through social support, diffusing information and reciprocities along with people’s social networks, and enhancing health norms and efficacy to facilitate health actions [27].

### Components of social capital

Social capital can be grouped into different types or components, depending on the criteria one uses to define the components. Structural considerations can lead to distinguishing networks with de facto many or few social interactions, tied and loose bonds, diverse or homogeneous members, high or low participation [28, 29]. In contrast, cognitive criteria may distinguish good or bad social interactions [28], feelings, values, attitudes and beliefs, as well as those attributed high or low reciprocity [30]. The commonly used indicators are trust and social support [31]. Any two types or components of social capital can influence health in different ways. Components defined according to cognitive criteria are primarily captured at the micro level and shape individuals’ behavioral norms through controlling health risk and provision of social help. Structural capital is on the other hand shaped by organization, institutions and culture which are more on the macro level [31, 32].

Cognitively and structurally defined social capital demonstrates different relations with people’s health and health behaviors [33–35]. In mental health, cognitive components showed strong evidence on disorders and contributed to better well-being. However, structural capital is much less beneficial and even demonstrated harmful consequences on mental health [30, 36]. A similar situation also appeared in health behaviors, with cognitively defined social capital protecting people from excessive drinking and cigarette smoking while structural components, on some occasions, may result in more drinking and smoking behaviors [34, 37]. Regarding HISB, we noticed that prior studies on health information seeking and social capital drew primary attention to structural components [24, 38, 39]. Social capital was estimated through group or community participation, as well as the Name Generator which centers on the instrumental resource embedded in social ties and fails in capturing cognitive social capital such as emotional support which is also valuable in health [40]. Besides, all these structural components showed positive association with people’s health information seeking, actual action or antecedents including self-efficacy and orientation.

Social capital can also be classified into bonding, bridging and linking social capital [41, 42]. In particular, the choice between bonding and bridging remains as one of the most critical distinctions [18, 43]. Bonding social capital is based on networks (therefore called bonding networks) in which people share similar social backgrounds, such as religious belief and social class [44]. People involved in bonding ties are highly homogeneous. Typical bonding ties are family relations or close-knit friends [31]. Bonding networks are intrinsically rich in providing emotional and instrumental support (refers to practical help, such as life caring and monetary support) [45]. At the same time, bonding capital can potentially be problematic [46–48], leading to exclusion of outsiders, excess claims on group members and restrictions on individual freedoms [49]. Bonding capital affects people’s health through psychological approaches [45]. It helps people maintain a sense of self-control [50], relieve stress [51] and enhance self-efficacy in performing certain health behaviors including HISB [38].

Bridging capital relies on more heterogeneous social networks (so called bridging networks) and often involves people from different social groups [44, 52, 53]. The heterogeneous bridging networks can provide individuals with a wider range of information support [45]. People can encounter others across different groups in bridging networks, and gather broader information as well as resources in dealing with health issues [38, 45].

We must assume that bridging and bonding networks affect HISB in different manners. However, the existing literature does not provide any conclusive evidence of this difference [38], also and especially for China, and particularly for HISB in China. Yet, there are studies that focused on other health aspects of bonding and bridging capital with relation to perceived general health and lifestyle behaviors in China. Not many differences emerged [54–56]. For mental health, there were negative or no effects of bridging in comparison with bonding capital [57, 58]. It recalls the aforementioned psychological value of strong bonding ties and implies that different consequences may be brought from bonding and bridging networks on HISB.

### Chinese culture

As briefly mentioned, the data for our analysis come from China. The reason for choosing China is the country’s unique cultural history. Strong social ties have traditionally been more firm than, for example, in Western cultures, and weak ties are found seldom only in China. If we map all individuals and their ties in the whole society, social structure in China can be visualized as a variety of dense clusters that scatter all over society but with very few external connections, and each cluster represents a social group [59, 60]. To this day, Chinese people still prefer to rely on close social relations instead of weak ones in their social life [60]. Besides, a strong tradition of familyism is ingrained in Chinese society [61]. Family ties are considered more trustworthy and reliable than ties in any other group an individual might join [62]. Family ties provide a feeling of security, unconditional protection and dependable obligations [63]. Chinese culture is moreover deeply formed by Confucianism, which tends to regulate individuals' behavior through social norms and emphasizes reciprocity in social contacts [64]. In spite of the import of social ties in Chinese culture, only a few studies on social capital have been conducted there.

Still, there is evidence from China also that social capital promotes self-perceived health status [58, 65] and life satisfaction [66, 67], as well as weakens feelings of loneliness [68] and depression [69, 70]. Social capital also encourages healthy diets and physical exercises [55, 56, 71], and it impedes alcohol consumption and cigarette smoking in China [55, 72, 73].

### Social capital and health information seeking

In the literature of social capital and HISB, Basu & Dutta (2008) found people with higher community participation reported higher levels of information orientation (indicating the willingness to seek health information) and efficacy (referring to respondents' perceived ability to seek health information they needed) [39]. In another study, social capital (measured by participation in a variety of social groups) was positively associated with health information seeking intention and self-efficacy, as well as scope of used information sources. Social capital also acted as a buffer attenuating negative impacts of poor health literacy on seeking intention and efficacy [38]. Still another study focused on real information-seeking behavior [24]. Authors found a positive relationship between social capital (indicated by the Name Generator) and the frequency of information seeking, usage of both personal and impersonal sources (internet, medical experts, family and friends), as well as source diversity. Results also showed that network size (measured by the number of alters in respondent’s networks) was positively associated with information seeking [24].

Apart from above-mentioned empirical studies that showed significant impact of structural social capital on HISB, several observations in the literature have also led our attention to social capital. First, trust in health information is often studied in health studies and higher trust in an information source predicts more frequent seeking behavior [74, 75]. Meanwhile, trust is one of the main concepts in social capital. Although trust in social capital refers to a more generalized trust in a group of people (e.g., trust in community or neighbors) or institutes that shares similar attributes (e.g., government institutes) [76], it is easy to image a correlation between a person’s’ general trust in an entity and his/her trust in health information from that entity. Second, people turn to the internet not only for finding health knowledge but also for social support, which again has been considered as a cognitive social capital component. For instance, patients seek emotional support from online health forums to cope with emotional distress caused by diseases [77]. The last observation coming from a traditional finding in communication research, which saw an inclination in people to communicate intensively in all (or many) channels. A person who watches a lot of health stories on TV will also read many health stories in the newspaper and talk much about health with friends and family. Generally, we expect persons who make use of one type of communication channel to be interested and use other channels as well.

On the other hand, Chinese people overall has stronger reliance on their social networks than people in the west [59, 60]. The traditional familyism culture emphasizes cohesion and connections between family members who serves as the center of bonding networks. Having interpersonal connections which can provides resources to the person is considered an essential factor in Chinese people’s social success [78], it somehow reflects the concept of norms of reciprocity in social capital. We expect, in the Chinese context, social capital will produce a impact on HISB. Based on our knowledge, there is no Chinese study that examined the association between social capital and HISB.

## Research questions

First, we are interested in social capital and its influence. The research question is: does social capital affect the intensity or frequency of HISB? (RQ 1). A second research question asks whether different components of social capital produce different reactions in the search for health information (RQ 2). The third question is concerned with turning to possible other antecedents of information seeking, which will demand other explanations (RQ 3).

## Method

### Sampling

The data used in this analysis originate from The Health Information National Trends Survey in China (HINTS-China), which was initially designed to understand Chinese people’s HISB and contains indicators reflecting individual social relations. Inspired by the U.S. Health Information National Trends Survey, China developed its own HINTS survey with a similar instrument structure. HINTS-China is a cross-sectional survey based on nationally representative samples. The first HINTS-China was administered in 2012, and the current one is from 2017, which adopted the same methodology. Data were collected in two Chinese cities: Beijing (the capital of China) and Hefei (a second-tier and capital city in Anhui Province). The target population was aged between 18 to 60 years [79]. In each city, respondents from urban and subsidiary rural areas were included. A multistage stratified random sampling technique was applied. According to the administrative division, each Chinese city typically consists of multiple districts in the urban area and multiple counties in the surrounding rural area. In Beijing and Hefei, a random rural county was elected, as was one urban district in each city. Sub-districts in each urban district and townships in each rural county were classified into three levels (high, medium and low) according to their economic development. At each economic level, a sub-district and a township were further randomly selected. Then smaller neighborhoods were randomly selected from each subdistrict or township. A certain number of households from neighborhood were randomly picked and one person from a household answered the questionnaire. Data was collected through door-to-door visits. Trained staff from The Chinese Center for Health Education visited sampled households with a print questionnaire. Respondents with sufficient literacy answered the questionnaire by themselves, while those who were unable to read or write were assisted by the trained staff. A more detailed survey methodology has been published by Zhao et al., (2015) [80]. A total of 3,090 adults aged from 18 to 60 years completed the survey.

### Measures

There were four measures for the dependent variables (HISB), Health information seeking, Cancer information seeking, Health information seeking from the internet, Health information seeking from traditional media. All asked frequencies as mentioned in the variable name. Answers to the first two questions were dichotomous (ever sought information on own initiative) with either yes (coded 1) or no (coded 0). The first two measures (health information seeking and cancer information seeking) tend to measure the incidences of seeking general health information and seeking information on a certain health topic, cancer in our case, among Chinese citizens. The prevalence of cancer has increased in Chinese populations, particularly among younger populations who have often been recognized as having lower risk of cancer [81]. Besides, ordinary populations are more likely to be aware of cancer than other diseases due to its chronic nature but generally high severity. The latter two measures asked how often respondents had been exposed to a number of communication channels, four traditional (health or medical information from newspapers, magazines, TV, and radio) and eight online sources including Web, News APP, medical health or food APP, other Apps, Baidu and other search engines, Microblog, WeChat, as well as Blog and forum. They fairly covered all relevant online and traditional media that Chinese persons used in daily life. By including both traditional media and the internet, we could capture potential differences between new and old media. Four-point frequency scales, ranging from never (= 1) to always (= 4) were used. Respondents’ answers were averaged as one index (traditional: α = 0.874; online: α = 0.903).

The independent variables included as measures of structural social capital were assessed separately with single items, inquiring about the number of people living in your current residence for bonding networks and the number of daily contacts for bridging networks (Table 1 for complete wording). We acknowledge that the single questions in both cases might fail to capture the picture adequately. A measure of bonding networks that should include very close friends. However, China still attaches significant importance to familyism [61]. Therefore, families’ ties play an essentially more important role in Chinese people’s bonding networks than friends’ do. Also, family members living in the same household are essential sources of social support [51]. Thus, we argue that the number of people who share the household with the respondent is still able to reflect a critical part of bonding networks.

**Table 1:** Overview of variables

Variable	Questionnaire	Scaling details
**Dependent variable (HISB**)		
Health information seeking	“Have you ever searched for health information on your own initiative?”	Single item, yes/no
Cancer information seeking	“Have you ever searched for cancer information on your own initiative?”	Same as above
Health information seeking from the internet	“Have you encountered health or medical information from [media source] in the past 12 months?”	4-category frequency scale, ranging from never (= 1) to always (= 4)
Health information seeking from traditional media	Similar to above	Same as above
**Independent variables: Social capital**		
Structural components		
Bonding networks	“How many people live in your current residence, including yourself?”	Single item
Bridging networks	“Apart from your family and relatives, how many people do you usually contact within a day?”	7-point scale was used ranging from None (= 1) to 100 or more persons (= 7)
Organization memberships	Number of community groups or organizations they are currently in	3-point scale
Cognitive components		
Emotional support	“When you need emotional support (e.g., need to discuss problems or make difficult decisions), is there anyone you can rely on?	Single item, yes/no or I am not sure
Informational support	Respondents have friends or family members to discuss health issues	Same as above
**Covariates**		
Trust in health information	“What’s your degree of trust in the health information provided by [media source]?”	24 items (= information sources), each rated by a 5-point scale from very untrustworthy (= 1) to very trustworthy (= 5)
Health information discussion	Frequency of discussing health-related issues with their family members or friends	Single item, 4 answer categories from 1 = never to 4 = always
Health information acquisition from organizations	If any joined organizations or groups can provide them health information	Single item, yes/no or I am not sure

The measure of bridging networks might contain very close friends which had better been counted as bonding. However, around half of the respondents answered that they usually contact more than 10 people (except for family members) within a day, and more than 20% of respondents even have contact with more than 20 people on a daily basis. Therefore, we consider the bridging networks as adequate also.

Organization memberships was used as another indicator to represent bridging social capital [57, 82] and can be characterized as a structural component [1].

Apart from structural social capital components, two cognitive components were included, emotional and health-related information support. The former asked respondents: whether they had anybody to rely on for emotional support. Information support inquired about respondents having friends or family to discuss health issues. We chose health as the focal information support as, unlike other topics such as travel, study or entertainment, discussing health issues requires a certain level of familiarity and intimacy. During the discussion of health issues, people gain advice and information from family members and friends [50].

Covariates of HISB were used as independent variables, mainly for control purposes to minimize confounding effects. Among these are Trust in health information from various sources such as websites, newspapers or family and friends. An exploratory factor analysis was conducted on the 24 trust items with orthogonal rotation (Varimax), see Additional file 1. for rotated factor loadings. Based on that, we retained five trust factors. They represented trust in health information from the internet (α = 0.903), traditional media (α = 0.877), interpersonal channels (α = 0.795), official institutes (α = 0.857), and informal organizations (α = 0.838) respectively.

Besides, two other variables provided information about respondents’ social networks and were heavily related to health information. Given that they somewhat deviate from the theoretical definition of social capital and reflect people’s HISB intention more, we decided to treat them as covariates also instead of social capital indicators. They are Health information discussion and Health information acquisition from organizations (Table 1).

We have included a series of socio-economic and-demographic variables to control the confounding effects. Details are shown in Table 2. Age was measured in years. Gender was represented by a dummy variable for female = 0 and male = 1. Education was measured as the highest grade completed from primary school and below (= 1) to bachelor degree above (= 6). Marital and occupation status were both dummy variables (1 = married, 0 = other; 1 = employed, 0 = retiree, student or the unemployed). Personal monthly income was categorized into eight groups with an 8-point scale from no income (= 1) to 10,000 Chinese yuan or above (= 8). Chronic diseases were also controlled as a dummy variable, and respondents without any listed chronic diseases were coded as 0. Residence was a dummy variable for rural (coded 0) and urban areas (coded 1).

**Table 2:** Descriptive statistics (unweighted, uncleaned)

Variables	*n* = 3090
Social-demographic	
Age (M/SD)	35.13/11.54
Gender (%)	
Female	61.1%
Male	38.9%
Education (%)	
Primary school and below	2.2%
Junior middle school	15.8%
High school	27.1%
Junior college	26.1%
Bachelor degree	23.1%
Bachelor degree above	5.7%
Marital status (%)	
Currently married	70.6%
Unmarried	29.4%
Employment (%)	
Employed	74.8%
Unemployed	25.2%
Personal income (%)	
Less than ¥ 1,500	16.5%
¥ 1,500–2,499	13%
¥ 2,500–4,999	40.9%
¥ 5,000–9,999	23.8%
¥ 10,000 and above	5.7%
Chronic diseases (%)	
Have	17.2%
Do not have	82.8%
Residence (%)	
Rural	50.8%
Urban	49.2%
Covariates of health information-seeking behavior	
Organizations providing health information (%)	
Yes	17.6%
No	82.4%
Health information discussion frequency (M/SD)	2.52/.83
Trusts in health information (M/SD)	
Internet	2.76/.77
Traditional media	2.91/.88
Interpersonal channels	3.86/.77
Official institutes	3.23/.94
Information organizations	2.62/.79
Social Capital	
Structure	
Bonding network (M/SD)	3.20/1.17
Bridging network (%)	
None	2.8%
1–4 persons	12.0%
5–9 persons	37.5%
10–19 persons	26.7%
20–49 persons	16.2%
50–99 persons	4.3%
100 or more persons	0.6%
Organization memberships (%)	
None	68.3%
A single organization	16.9%
Two or more organizations	14.7%
Cognitive	
Emotional support (%)	
Yes	85.6%
No	14.4%
Information support (%)	
Yes	73.5%
No	26.5%
Health information-seeking behavior	
Health information seeking (%)	
Yes	31.3%
No	68.7%
Cancer information seeking (%)	
Yes	16.9%
No	83.1%
Health information seeking from the internet (M/SD)	2.12/.70
Health information seeking from traditional media (M/SD)	2.01/.76

### Statistical analysis

All statistical analyses were operated in SPSS version 26. We first used Cronbach’s alpha coefficient to evaluate the internal consistency and reliability of all scales. Besides, exploratory factor analysis (EFA) was conducted to understand underlying structure of the original trust index in health information, which generated five trust factors: trust in health information from the internet, traditional media, interpersonal channels, official institutes, and informal organizations. Hierarchical multiple regression analyses and binary logistic regression tests were operated to investigate the relationship between social capital and HISB indicators. Before the final analysis began, the dataset was weighted due to the overrepresentation of female respondents (61.1%). The percentage of females in the weighted data set corresponds to the female proportion in the entire country, as should be (48.8%) according to the Seventh National Census.^1^ Outliers were cleaned before running inferential statistics, regressions in our study, to improve the statistical power. We found in bonding networks 17 respondents had seven or more people (including themselves) living in his/her residence and others all answered less than seven. Therefore we decided to treat these seventeen people as outliers accounting for 0.6% (17 out of 3090) of the total sample. We used a 95% confidence level for the confidence interval (CI) in all analyses.

## Results

The descriptive statistics are presented in Table 2. The major independent variable, social capital was operationalized in five indicators. The average bonding network size (family who shared living quarters) was 3.20 with a standard deviation of 1.17. In bridging networks, 47.8% of residents have daily contact with more than 9 people, and in particular, 4.9% of respondents said that they usually meet more than 49 people every day. However, 2.8% (87 out of 3090) people had no external contacts apart from family ties. Concerning group memberships, a large part of people (68.3%) had not joined any organization, 16.9% of them reported membership in a single organization, and the rest took part in multiple groups. As to social support, the majority of respondents (85.6%) believed they had someone to rely on when emotional support was needed, and 73.5% of people answered that they had family members or friends to discuss health issues (information support).

Concerning the dependent variable HISB, only 31.3% of participants have ever searched health information on their own initiative, even less (16.9%) had searched for cancer information. Comparing with traditional media (the mean value is 2.01 with a standard deviation of 0.76), people encounter health information more through the internet (the mean value is 2.12 with a standard deviation of 0.70).

Table 3 presents the results of binary logistic regression tests of two HISB dichotomous variables, as well as multiple linear regressions of health information seeking on the internet and traditional media, which were available as scales.

**Table 3:** Binary logistic regression and multiple linear regression of health information-seeking behavior (weighted, cleaned)

Variables	Health information seeking	Cancer information seeking	Seeking from the internet	Seeking from traditional media
	Model 1	Model 2	Model 3	Model 4
Social-demographic				
Age	1.020*** (1.008,1.032)	.996 (.983,1.010)	-.009***	.014***
Gender(male)	1.117 (.937,1.330)	1.203 (.974,1.486)	-.060*	-.027
Education	.990 (.904,1.083)	.894* (.803,1.022)	.054***	.009
Marital status(married)	1.147 (.899,1.480)	.887 (.659,.1.194)	-.007	-.028
Employment(employed)	.773 (.579,1.033)	1.458* (1.021, 2.084)	.047	-.032
Personal income	.970 (.908,1.037)	.890** (.822,.964)	-.007	-.001
Chronic diseases(have)	1.859*** (1.485,2.326)	1.517** (1.170, 1.968)	.079*	.122***
Urban or rural(urban)	1.489*** (1.237,1.792)	1.411** (1.128,1.766)	.136***	.160***
Covariates of health information-seeking behavior				
Organizations providing health information	1.522*** (1.173,1.974)	2.208*** (1.654,2.946)	.068	.093*
Health information discussion frequency	1.614*** (1.433,1.827)	1.816** (1.566,2.105)	.079***	.098***
Trusts in health information				
Internet	1.710*** (1.447, 2.021)	1.473*** (1.209,1.795)	.288***	.054*
Traditional media	1.090 (.954,1.245)	1.370*** (1.167,1.609)	.078***	.286***
Interpersonal channels	1.204** (1.055,1.375)	1.046 (.894, 1.224)	-.023	-.044*
Official institutes	1.127* (1.002,1.267)	1.096 (.952, 1.262)	.045**	.015
Informal organizations	.753*** (.646,.877)	.729*** (.610,.871)	-.103***	-.024
Social capital				
Structural				
Bonding networks	1.061 (.981,1.147)	1.091 (.994, 1.197)	.001	.002
Bridging networks	1.041 (.965,1.122)	1.014 (.927,1.110)	.048***	.050***
Organization memberships	1.121 (.973,1.292)	1.221* (1.038,1.434)	.084***	.063**
Cognitive				
Emotional support	.657*** (.511,.845)	.613*** (.460,.818)	-.106**	-.092**
Information support	1.564*** (1.233,1.983)	1.091 (.814,1.461)	.012	-.033
R^2^_adjusted_			.190	.241

^***^P ≤ 0.001

**P ≤ 0.01

*P ≤ 0.05

Five indicators for social capital as the independent variable were combined in a brief look at suitable bivariate analyses with four measures of information seeking as the dependent variable. Of 20 relationships, half showed significant differences from 0. The strength of bridging networks was positively associated with use of the internet (β = 0.048, *P* ≤ 0.001) and traditional media (β = 0.050, *P* ≤ 0.001) to seek health information. Besides, the finding for organizations encouraged Chinese went along with searching for cancer information (OR = 1.221, *P* ≤ 0.05), to seek information through old and new media (the internet: β = 0.084, *P* ≤ 0.001; traditional media: β = 063, *P* ≤ 0.01). The analysis also provides results in quite different directions: bonding networks remains insignificant.

Comparing the cognitive division, the results were clear-cut. Emotional support was constantly associated with all HISB variables in a negative way. So people who have someone to rely on when facing life difficulties are less likely to search for health information (OR = 0.657, *P* ≤ 0.001), cancer information (OR = 0.613, *P* ≤ 0.001), seeking through the internet and traditional media (β = -0.106, *P* ≤ 0.01 respectively β = -0.092, *P* ≤ 0.01). Information support only demonstrated a positive relation with health information seeking (OR = 1.564, *P* ≤ 0.001), while remaining insignificant with the other three HISB indicators.

Stopping here to look back for a short moment, we can say people with many or stronger bridging social connections search the internet more often than other people do, but the same is true of traditional media channels. The higher attention paid to the potentials of the new information device is not contingent on whether the channel is new or has been around for a while, and the attention difference is displayed only if the comparison is made for the bridging rather than the bonding component of peoples’ social networks. A wide array of results confirm that bridging social capital components in general do matter when antecedents of the search behavior are wanted [38, 39]. RQ 1 receives some answer expressed in the form: “yes, but not everywhere.” So does RQ2 when it is found that people who have strong emotional support do not necessarily go out and find health or cancer information on their own. The accessibility of information sources does not make a difference that emotionally supported people were less use both traditional media and the internet to get health information.

The attention was also paid to trust in health information, we found they generally promoted Chinese people’s HISB, except for trust in informal organizations which constantly showed negatively association with HISB and trust in interpersonal channels that negatively correlated to traditional media use. Trust in the internet health information appeared as the most significant predictor, which showed positive association with all HISB variables.

## Discussion and conclusion

This study examined the association between social capital and HISB including general health information seeking, cancer information seeking, and the frequency of using the internet and traditional media as information sources in Chinese populations. We found that social capital, especially structural components, generally entices Chinese people to adopt HISB, in which bridging ties are more promotive than bonding ones; on the other hand, cognitive components of emotional support appeared as the only negative predictor that damages Chinese people’s interest in seeking for health and cancer information. It also impeded people from using the internet and traditional media to get health information. Below, we highlight three major findings on social capital that contribute to the existing literature.

First, our study, aligning with previous evidence, confirmed structural social capital, including networks and group memberships promotes HISB [24, 38, 39]. Exposure to health information may drive other members (apart from active seekers) inside the network to search for health information due to peer pressure or enhanced social norms of health [39]. As shown in the current study, we found group memberships positively associated with all health information-seeking indicators regardless whether the organization can provide them with health information. We also found denser bridging networks associated with more actively searching for health and cancer information.

Second, we found a significant difference between bonding and bridging connections. Family members and close friends (namely bonding relations) are often consulted first when a person faces health issues, they provide assistance that helps handle tough situations [51]. These social ties serve as information sources that provide health information as well as a validation tool to encourage people to search for relevant health information, so that the person can better cope with difficulties [83]. However, bonding networks did not show any significant results in the current study. It might because the data was not collected among people facing difficulties such as cancer patients, the psychological value of bonding ties were not captured. Bridging networking on the other hand promotes HISB in our study, as it can open wide ranges of information and intrinsically rich in information support [44, 45]. Chinese residents with denser and more diverse bridging connections are thus more likely to come across health information, which may awake their health awareness and further encourage health information seeking behavior. People with more bridging capital tend to have better higher socio-economic [84]. They are more aware of health and being active in seeking relevant information for their health. However, the impact of bridging ties remained significant after controlling several socio-economic indicators including education, occupation status, personal income and residence (rural versus urban). This independent influence of bridging ties, regardless of socio-economic, should come from the nature of bridging networks.

Lastly, literature usually suggests that social support could improve people’s capacity in finding and understanding health information [51]. Emotional support can practically improve people’ self-esteem and self-confidence that help cope with personal limitations [51]. However, our study surprisingly found significant negative relations between emotional support and all HISB indicators. This was already interpreted by Shaw and his colleagues (2008) who found individuals perceived to be with a worse condition including lacking social support are more likely to search health information online. The reason is the person surrounded by strong supportive social relations such as family and close friends might not realize the necessity to gather information from impersonal media. Instead, they tend to rely on their personal networks. Therefore, such a relation between media and interpersonal venues become complementary. A few previous studies, though, have also shown low social support predicts more active HISB [85, 86]. Considering that Chinese people heavily rely on strong ties and attach great importance to the concept of familyism [60, 62], the person who has emotional support in China might have a stronger sense of dependency than their counterparts in Western societies. This strong feeling of having someone to rely on might explain the negative relation between emotional support and HISB in China. We call for future research to better understand underlying mechanisms of this negative association. Besides, only little difference found between new and old media that Chinese people’s social relations do not affect their choice of different impersonal media for health information.

In addition to social capital, trust in information source has significant impact on health information seeking. Particularly, trust in internet health information promotes all kinds of HISB in China including general health information, cancer information seeking, both health information seeking on the internet and traditional media. It appeared as a universal promotor of HISB regardless of media type and topic of information. However, health information trust is too narrow compared with trust measures used in social capital studies and it is determined by national culture [87]. Therefore, we call for future studies which apply trust measures originated from social capital realm and based on different culture contexts to better understand the impact of trust.

Be aware that the current study only reflects impacts of social capital on general population’s HISB. The results may not apply to patients with special health conditions which have strong social stigma attached with such as mental disorders or sexually transmitted diseases [88, 89]. Patients with these condition are fear to seek medical help in China [90], thus their HISB can differ from normal populations and leads to inapplicability of our study results.

This study presents a major advance as the first empirical study that draws attention to Chinese people’s social capital and their health information seeking behavior. It showed the distinguished consequences of multiple social capital components on individual’ health information seeking. Nevertheless, it also has limitations. As a multidimensional concept, social capital can be measured from different perspectives [45]. No agreement has been achieved in terms of how to measure it, which imposes one of the biggest challenges to social capital researchers [91]. Except for commonly used measures such as trust, organization participation and social support, many studies used their own measures such as the feeling of community [92] or price of gifts for the elderly in the family [93]. Our study also missed measures of a main social capital concept: trust. Despite trust in health information were included, they are too narrative and deviate from trust measures commonly used in social capital literature [18]. Besides, the current study solely looked at individual-level social capital, while social capital is often conceptualized at both individual and collective levels [19, 94]. It would be ideal to include the collective-level social capital in our study, such neighbor-level social support. We also used a self-report single question to measure each component of social capital, it might lose power in detecting respondents’ real levels of social capital and result in justification bias and misclassifications.

For future social capital studies in China, we noticed that there are many health conditions (e.g., cancer or diabetes mortality, obesity, infectious diseases, mental health and so on) which have been explored in the western contexts but remain underestimated in China. Taking sexually transmitted diseases as an example, significant correlations between social capital and HIV infections were found in the western population [35, 95], however, we rarely know in Chinese populations. Thus, we suggest future Chinese studies expand attention to health conditions that have not been studied in China while having significant impacts on public health.

## Supplementary Information

**Additional file 1.** Exploratory factor analysis of trusts in health information: rotated factor loadings.

## Data Availability

The datasets used and/or analysed during the current study are available from the corresponding author on reasonable request.

## Abbreviations

HISB: Health information-seeking behavior
HINTS-China: Health Information National Trends Survey – China

## Footnotes

1 The detailed information about the Seventh National Census is announced in http://www.stats.gov.cn/english/
