# Supplementary material for: Social capital and health information seeking in China
Source: BMC Public Health. 2022 Aug 10;22:1525. doi: 10.1186/s12889-022-13895-2 (PMC9364581; doi:10.1186/s12889-022-13895-2)
Supplement: Supplementary file 1 — Additional file 1. Exploratory factor analysis of trusts in health information: rotated factor loadings. [file 12889_2022_13895_MOESM1_ESM.docx]

# Additional file 1

Exploratory factor analysis of trusts in health information: rotated factor loadings.

| Item  *“What’s your degree of trust in the health information provided by [media source]?”* | The internet | Traditional media | Informal organizations | Official institutes | Interpersonal channels |
| --- | --- | --- | --- | --- | --- |
| Doctor or health specialist |  |  |  |  | **.712** |
| Family member |  |  |  |  | **.884** |
| Friend or colleague |  |  |  |  | **.834** |
| Newspaper |  | **.785** |  |  |  |
| Magazine |  | **.777** |  |  |  |
| Television |  | **.725** |  |  |  |
| Radio |  | **.715** |  |  |  |
| Book |  | .537 |  | .533 |  |
| Official government agency |  | .312 |  | **.763** |  |
| International organization |  |  |  | **.792** |  |
| Academic research institution |  |  |  | **.796** |  |
| Business organization |  |  | **.682** |  |  |
| Religious organization or leader |  |  | **.783** |  |  |
| Community or neighborhood committee |  |  | **.579** |  |  |
| Charitable organization | .336 |  | **.665** |  |  |
| Telephone hotline | .388 |  | **.671** |  |  |
| Website | **.615** |  | .356 |  |  |
| News APP | **.654** |  |  |  |  |
| Medical health or food APP | **.639** |  |  |  |  |
| Other APP | **.664** |  |  |  |  |
| Baidu and other search engines | **.732** |  |  |  |  |
| MicroBlog | **.756** |  |  |  |  |
| WeChat | **.673** |  |  |  |  |
| Blog and forum | **.759** |  |  |  |  |
| Eigenvalues | 4.646 | 3.347 | 3.080 | 2.796 | 2.315 |
| % of variance | 19.359 | 13.947 | 12.833 | 11.650 | 9.647 |
| α | .903 | .877 | .838 | .857 | .795 |

Eigenvalues smaller than 0.30 are suppressed; Items with the bold eigenvalue represent the factor it subordinated to.
